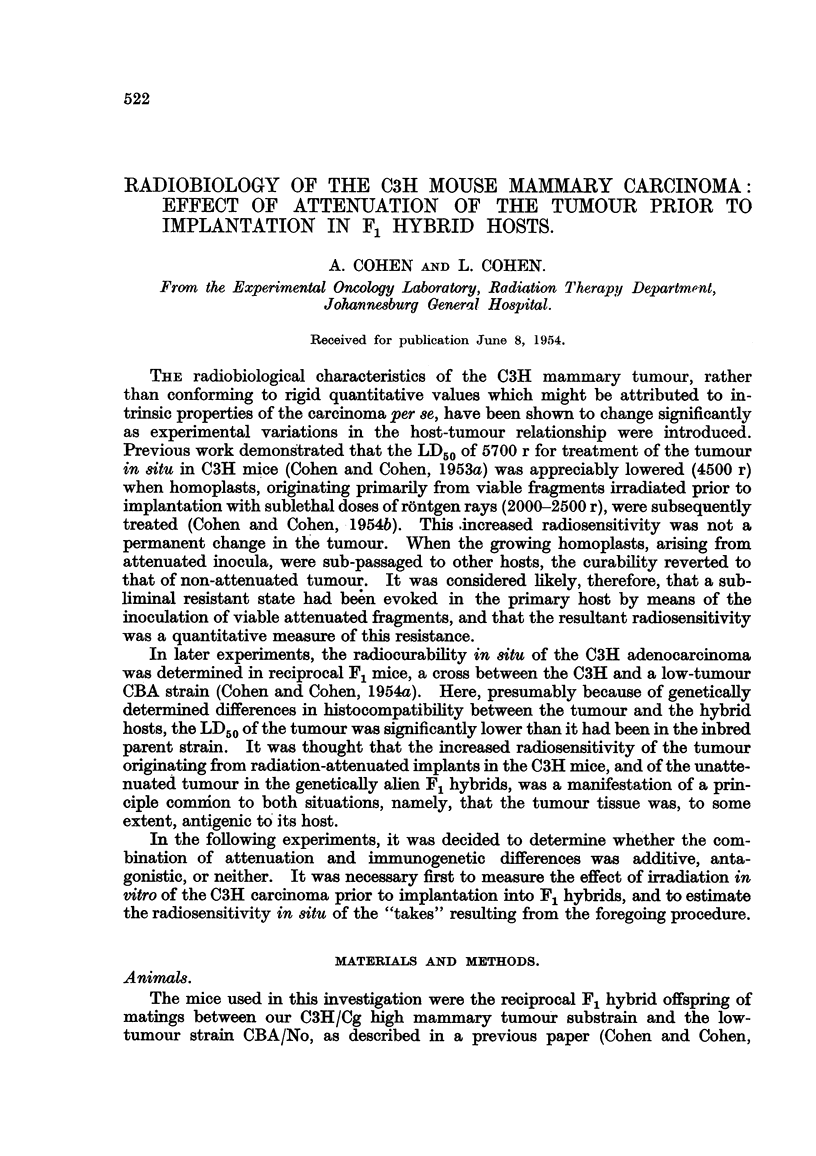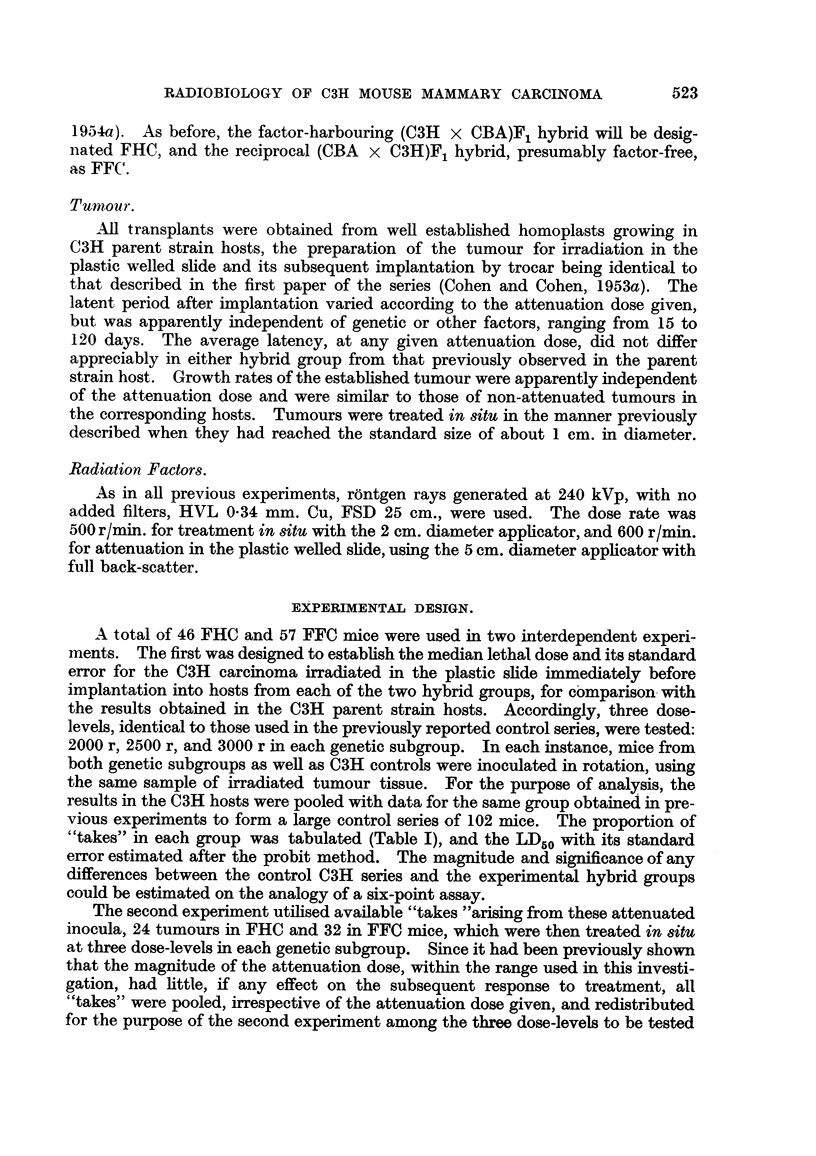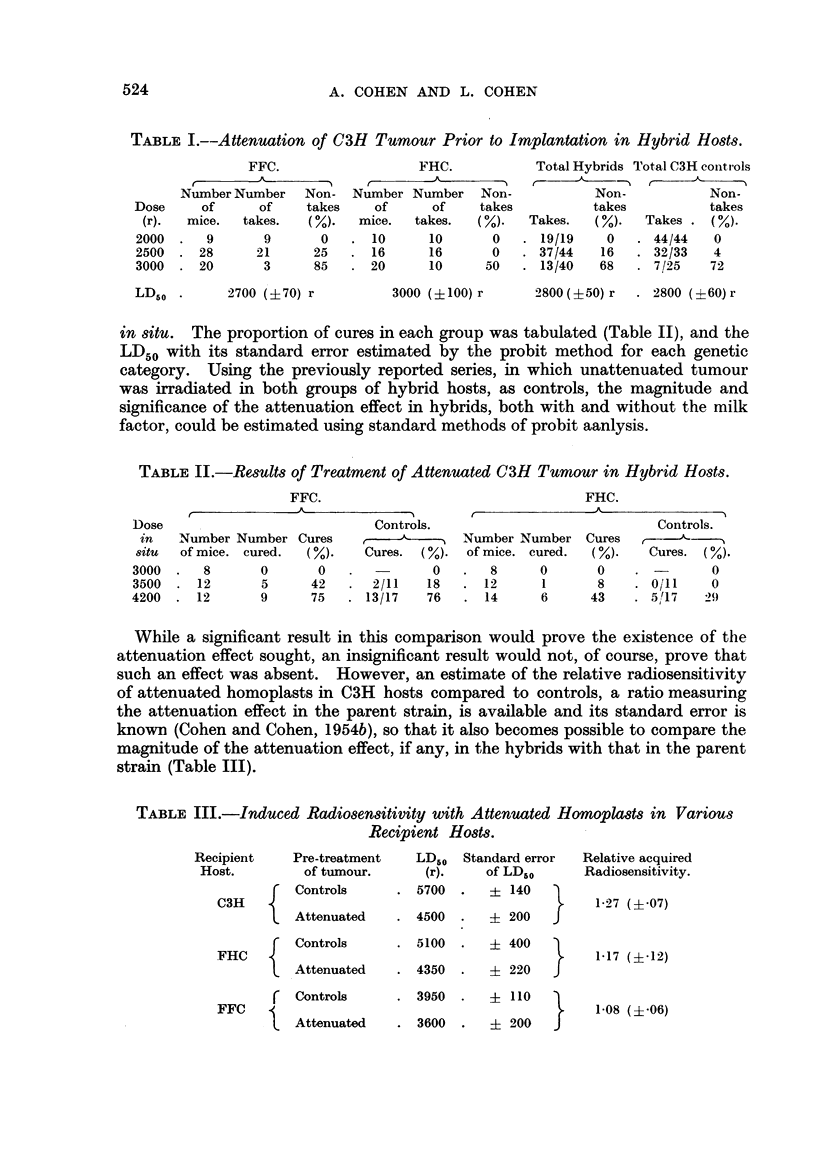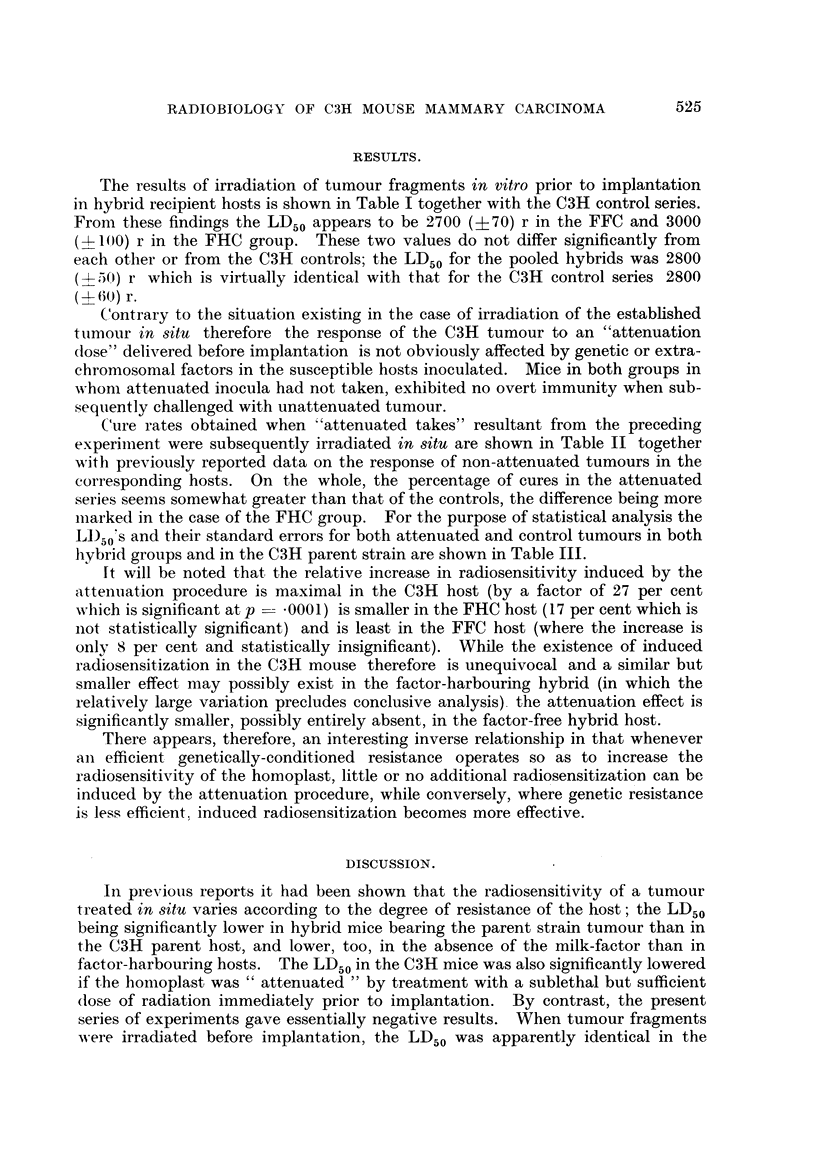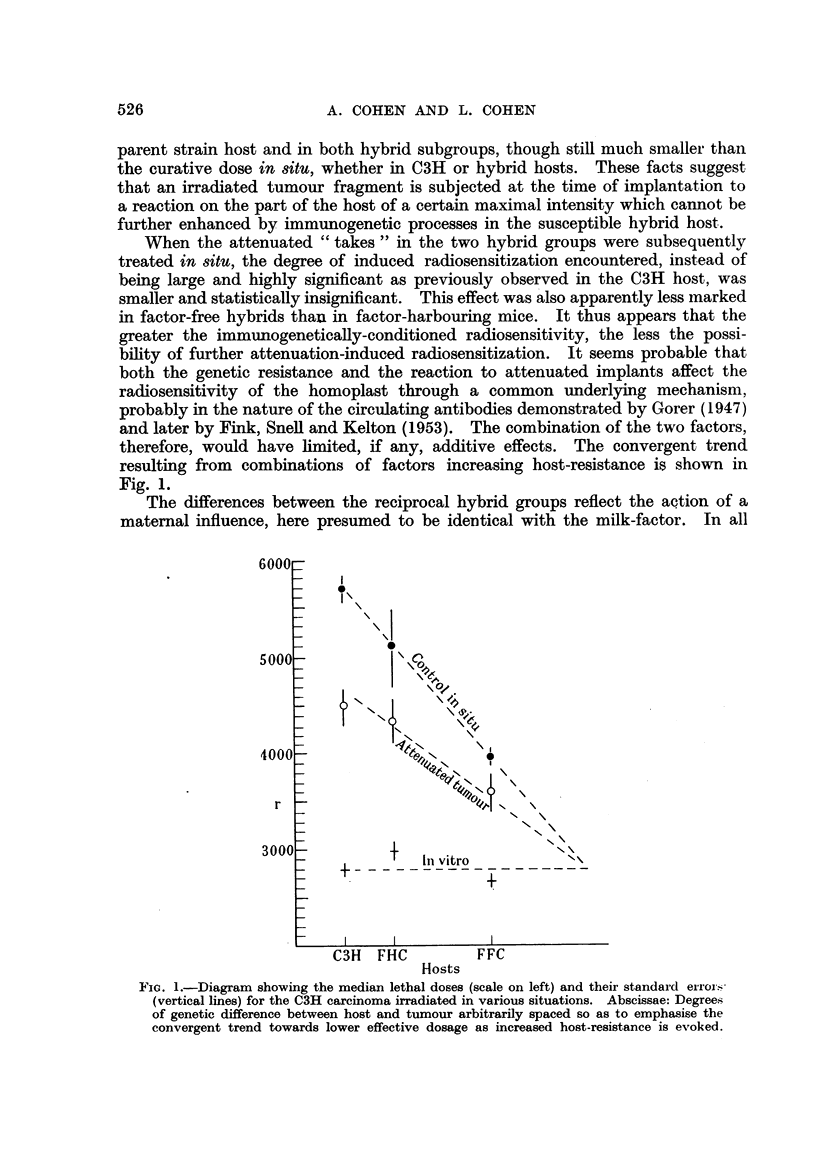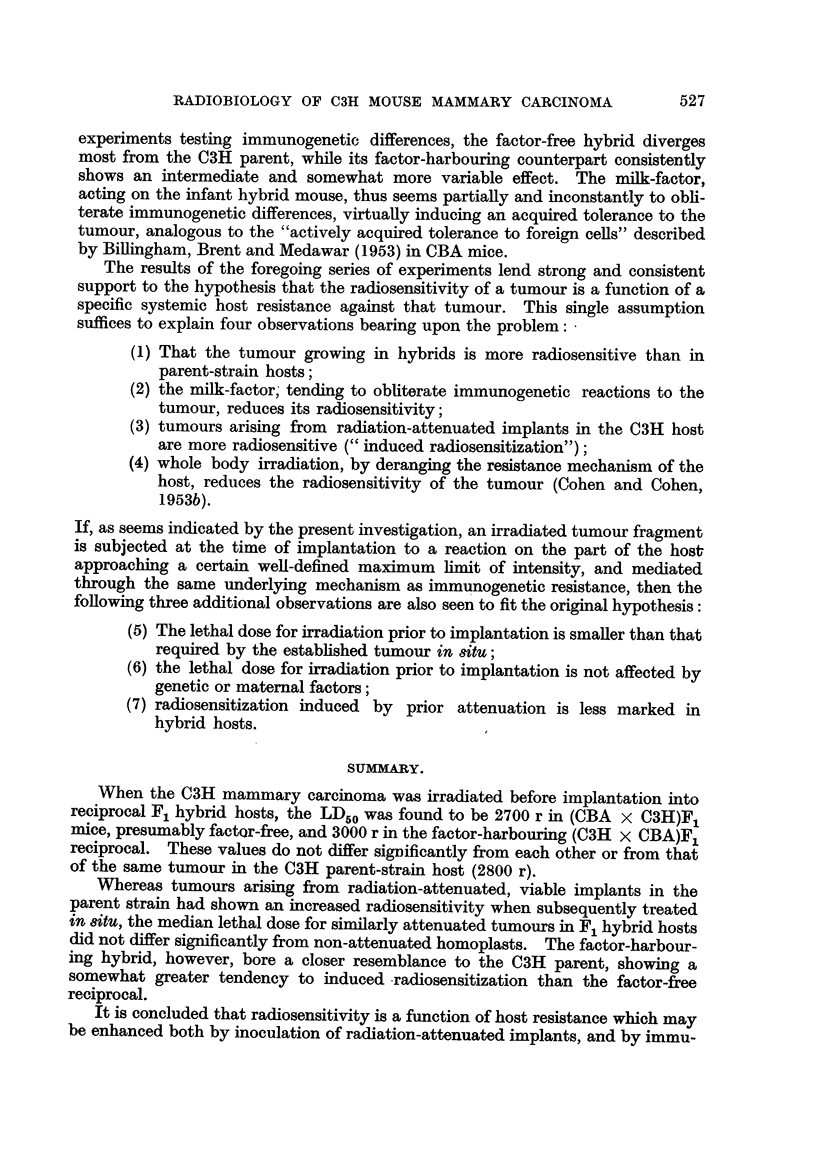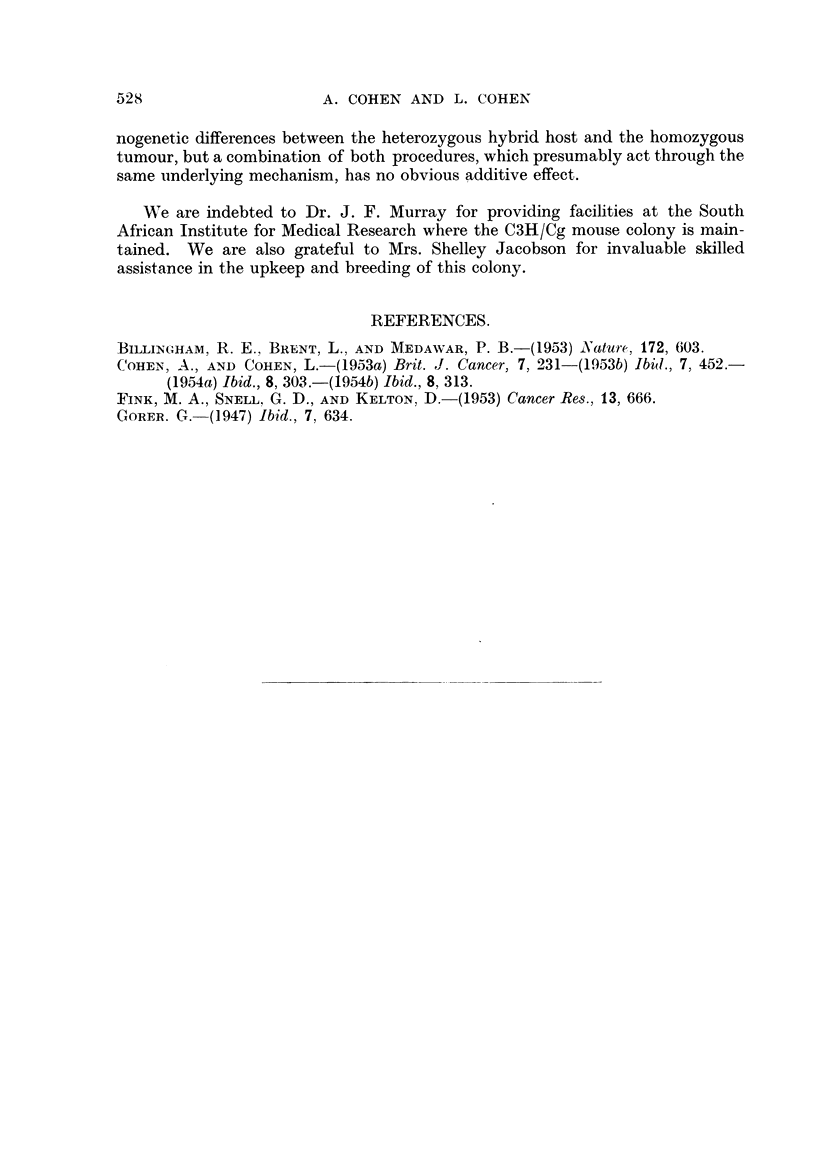# Radiobiology of the C3H Mouse Mammary Carcinoma: Effect of Attenuation of the Tumour Prior to Implantation in F_1_ Hybrid Hosts

**DOI:** 10.1038/bjc.1954.55

**Published:** 1954-09

**Authors:** A. Cohen, L. Cohen


### RADIOBIOLOGY OF THE C3H MOUSE MAMMARY CARCINOMA: EFFECT OF ATTENUATION OF THE TUMOUR PRIOR TO IMPLANTATION IN $F_1$ HYBRID HOSTS.

A. COHEN AND L. COHEN.

*From the Experimental Oncology Laboratory, Radiation Therapy Department, Johannesburg General Hospital.*

Received for publication June 8, 1954.

THE radiobiological characteristics of the C3H mammary tumour, rather than conforming to rigid quantitative values which might be attributed to intrinsic properties of the carcinoma *per se*, have been shown to change significantly as experimental variations in the host-tumour relationship were introduced. Previous work demonstrated that the $LD_{50}$ of 5700 r for treatment of the tumour *in situ* in C3H mice (Cohen and Cohen, 1953*a*) was appreciably lowered (4500 r) when homoplasts, originating primarily from viable fragments irradiated prior to implantation with sublethal doses of röntgen rays (2000–2500 r), were subsequently treated (Cohen and Cohen, 1954*b*). This increased radiosensitivity was not a permanent change in the tumour. When the growing homoplasts, arising from attenuated inocula, were sub-passaged to other hosts, the curability reverted to that of non-attenuated tumour. It was considered likely, therefore, that a subliminal resistant state had been evoked in the primary host by means of the inoculation of viable attenuated fragments, and that the resultant radiosensitivity was a quantitative measure of this resistance.

In later experiments, the radiocurability *in situ* of the C3H adenocarcinoma was determined in reciprocal $F_1$ mice, a cross between the C3H and a low-tumour CBA strain (Cohen and Cohen, 1954*a*). Here, presumably because of genetically determined differences in histocompatibility between the tumour and the hybrid hosts, the $LD_{50}$ of the tumour was significantly lower than it had been in the inbred parent strain. It was thought that the increased radiosensitivity of the tumour originating from radiation-attenuated implants in the C3H mice, and of the unattenuated tumour in the genetically alien $F_1$ hybrids, was a manifestation of a principle common to both situations, namely, that the tumour tissue was, to some extent, antigenic to its host.

In the following experiments, it was decided to determine whether the combination of attenuation and immunogenetic differences was additive, antagonistic, or neither. It was necessary first to measure the effect of irradiation *in vitro* of the C3H carcinoma prior to implantation into $F_1$ hybrids, and to estimate the radiosensitivity *in situ* of the "takes" resulting from the foregoing procedure.

### MATERIALS AND METHODS.

### *Animals.*

The mice used in this investigation were the reciprocal $F_1$ hybrid offspring of matings between our C3H/Cg high mammary tumour substrain and the low-tumour strain CBA/No, as described in a previous paper (Cohen and Cohen,

1954*a*). As before, the factor-harbouring (C3H × CBA)$F_1$ hybrid will be designated FHC, and the reciprocal (CBA × C3H)$F_1$ hybrid, presumably factor-free, as FFC.

*Tumour.*

All transplants were obtained from well established homoplasts growing in C3H parent strain hosts, the preparation of the tumour for irradiation in the plastic welled slide and its subsequent implantation by trocar being identical to that described in the first paper of the series (Cohen and Cohen, 1953*a*). The latent period after implantation varied according to the attenuation dose given, but was apparently independent of genetic or other factors, ranging from 15 to 120 days. The average latency, at any given attenuation dose, did not differ appreciably in either hybrid group from that previously observed in the parent strain host. Growth rates of the established tumour were apparently independent of the attenuation dose and were similar to those of non-attenuated tumours in the corresponding hosts. Tumours were treated *in situ* in the manner previously described when they had reached the standard size of about 1 cm. in diameter.

*Radiation Factors.*

As in all previous experiments, röntgen rays generated at 240 kVp, with no added filters, HVL 0·34 mm. Cu, FSD 25 cm., were used. The dose rate was 500 r/min. for treatment *in situ* with the 2 cm. diameter applicator, and 600 r/min. for attenuation in the plastic welled slide, using the 5 cm. diameter applicator with full back-scatter.

### EXPERIMENTAL DESIGN.

A total of 46 FHC and 57 FFC mice were used in two interdependent experiments. The first was designed to establish the median lethal dose and its standard error for the C3H carcinoma irradiated in the plastic slide immediately before implantation into hosts from each of the two hybrid groups, for comparison with the results obtained in the C3H parent strain hosts. Accordingly, three dose-levels, identical to those used in the previously reported control series, were tested: 2000 r, 2500 r, and 3000 r in each genetic subgroup. In each instance, mice from both genetic subgroups as well as C3H controls were inoculated in rotation, using the same sample of irradiated tumour tissue. For the purpose of analysis, the results in the C3H hosts were pooled with data for the same group obtained in previous experiments to form a large control series of 102 mice. The proportion of "takes" in each group was tabulated (Table I), and the $LD_{50}$ with its standard error estimated after the probit method. The magnitude and significance of any differences between the control C3H series and the experimental hybrid groups could be estimated on the analogy of a six-point assay.

The second experiment utilised available "takes "arising from these attenuated inocula, 24 tumours in FHC and 32 in FFC mice, which were then treated *in situ* at three dose-levels in each genetic subgroup. Since it had been previously shown that the magnitude of the attenuation dose, within the range used in this investigation, had little, if any effect on the subsequent response to treatment, all "takes" were pooled, irrespective of the attenuation dose given, and redistributed for the purpose of the second experiment among the three dose-levels to be tested

TABLE I.—*Attenuation of C3H Tumour Prior to Implantation in Hybrid Hosts.*

| | FFC. | | | FHC. | | | Total Hybrids | | Total C3H controls | |
|---|---|---|---|---|---|---|---|---|---|---|
| Dose (r). | Number of mice. | Number of takes. | Non-takes (%). | Number of mice. | Number of takes. | Non-takes (%). | Takes. | Non-takes (%). | Takes. | Non-takes (%). |
| 2000 | 9 | 9 | 0 | 10 | 10 | 0 | 19/19 | 0 | 44/44 | 0 |
| 2500 | 28 | 21 | 25 | 16 | 16 | 0 | 37/44 | 16 | 32/33 | 4 |
| 3000 | 20 | 3 | 85 | 20 | 10 | 50 | 13/40 | 68 | 7/25 | 72 |
| $LD_{50}$ | 2700 (±70) r | | | 3000 (±100) r | | | 2800 (±50) r | | 2800 (±60) r | |

*in situ.* The proportion of cures in each group was tabulated (Table II), and the $LD_{50}$ with its standard error estimated by the probit method for each genetic category. Using the previously reported series, in which unattenuated tumour was irradiated in both groups of hybrid hosts, as controls, the magnitude and significance of the attenuation effect in hybrids, both with and without the milk factor, could be estimated using standard methods of probit aanlysis.

TABLE II.—*Results of Treatment of Attenuated C3H Tumour in Hybrid Hosts.*

| | FFC. | | | | | FHC. | | | | |
|---|---|---|---|---|---|---|---|---|---|---|
| | | | | Controls. | | | | | Controls. | |
| Dose *in situ* | Number of mice. | Number cured. | Cures (%). | Cures. | (%). | Number of mice. | Number cured. | Cures (%). | Cures. | (%). |
| 3000 | 8 | 0 | 0 | — | 0 | 8 | 0 | 0 | — | 0 |
| 3500 | 12 | 5 | 42 | 2/11 | 18 | 12 | 1 | 8 | 0/11 | 0 |
| 4200 | 12 | 9 | 75 | 13/17 | 76 | 14 | 6 | 43 | 5/17 | 29 |

While a significant result in this comparison would prove the existence of the attenuation effect sought, an insignificant result would not, of course, prove that such an effect was absent. However, an estimate of the relative radiosensitivity of attenuated homoplasts in C3H hosts compared to controls, a ratio measuring the attenuation effect in the parent strain, is available and its standard error is known (Cohen and Cohen, 1954*b*), so that it also becomes possible to compare the magnitude of the attenuation effect, if any, in the hybrids with that in the parent strain (Table III).

TABLE III.—*Induced Radiosensitivity with Attenuated Homoplasts in Various Recipient Hosts.*

| Recipient Host. | Pre-treatment of tumour. | $LD_{50}$ (r). | Standard error of $LD_{50}$ | Relative acquired Radiosensitivity. |
|---|---|---|---|---|
| C3H | Controls | 5700 | ± 140 | 1·27 (±·07) |
| | Attenuated | 4500 | ± 200 | |
| FHC | Controls | 5100 | ± 400 | 1·17 (±·12) |
| | Attenuated | 4350 | ± 220 | |
| FFC | Controls | 3950 | ± 110 | 1·08 (±·06) |
| | Attenuated | 3600 | ± 200 | |

### RESULTS.

The results of irradiation of tumour fragments *in vitro* prior to implantation in hybrid recipient hosts is shown in Table I together with the C3H control series. From these findings the $LD_{50}$ appears to be 2700 ($\pm$70) r in the FFC and 3000 ($\pm$100) r in the FHC group. These two values do not differ significantly from each other or from the C3H controls; the $LD_{50}$ for the pooled hybrids was 2800 ($\pm$50) r which is virtually identical with that for the C3H control series 2800 ($\pm$60) r.

Contrary to the situation existing in the case of irradiation of the established tumour *in situ* therefore the response of the C3H tumour to an "attenuation dose" delivered before implantation is not obviously affected by genetic or extra-chromosomal factors in the susceptible hosts inoculated. Mice in both groups in whom attenuated inocula had not taken, exhibited no overt immunity when subsequently challenged with unattenuated tumour.

Cure rates obtained when "attenuated takes" resultant from the preceding experiment were subsequently irradiated *in situ* are shown in Table II together with previously reported data on the response of non-attenuated tumours in the corresponding hosts. On the whole, the percentage of cures in the attenuated series seems somewhat greater than that of the controls, the difference being more marked in the case of the FHC group. For the purpose of statistical analysis the $LD_{50}$'s and their standard errors for both attenuated and control tumours in both hybrid groups and in the C3H parent strain are shown in Table III.

It will be noted that the relative increase in radiosensitivity induced by the attenuation procedure is maximal in the C3H host (by a factor of 27 per cent which is significant at $p = \cdot 0001$) is smaller in the FHC host (17 per cent which is not statistically significant) and is least in the FFC host (where the increase is only 8 per cent and statistically insignificant). While the existence of induced radiosensitization in the C3H mouse therefore is unequivocal and a similar but smaller effect may possibly exist in the factor-harbouring hybrid (in which the relatively large variation precludes conclusive analysis). the attenuation effect is significantly smaller, possibly entirely absent, in the factor-free hybrid host.

There appears, therefore, an interesting inverse relationship in that whenever an efficient genetically-conditioned resistance operates so as to increase the radiosensitivity of the homoplast, little or no additional radiosensitization can be induced by the attenuation procedure, while conversely, where genetic resistance is less efficient, induced radiosensitization becomes more effective.

### DISCUSSION.

In previous reports it had been shown that the radiosensitivity of a tumour treated *in situ* varies according to the degree of resistance of the host; the $LD_{50}$ being significantly lower in hybrid mice bearing the parent strain tumour than in the C3H parent host, and lower, too, in the absence of the milk-factor than in factor-harbouring hosts. The $LD_{50}$ in the C3H mice was also significantly lowered if the homoplast was " attenuated " by treatment with a sublethal but sufficient dose of radiation immediately prior to implantation. By contrast, the present series of experiments gave essentially negative results. When tumour fragments were irradiated before implantation, the $LD_{50}$ was apparently identical in the parent strain host and in both hybrid subgroups, though still much smaller than the curative dose *in situ*, whether in C3H or hybrid hosts. These facts suggest that an irradiated tumour fragment is subjected at the time of implantation to a reaction on the part of the host of a certain maximal intensity which cannot be further enhanced by immunogenetic processes in the susceptible hybrid host.

When the attenuated " takes " in the two hybrid groups were subsequently treated *in situ*, the degree of induced radiosensitization encountered, instead of being large and highly significant as previously observed in the C3H host, was smaller and statistically insignificant. This effect was also apparently less marked in factor-free hybrids than in factor-harbouring mice. It thus appears that the greater the immunogenetically-conditioned radiosensitivity, the less the possibility of further attenuation-induced radiosensitization. It seems probable that both the genetic resistance and the reaction to attenuated implants affect the radiosensitivity of the homoplast through a common underlying mechanism, probably in the nature of the circulating antibodies demonstrated by Gorer (1947) and later by Fink, Snell and Kelton (1953). The combination of the two factors, therefore, would have limited, if any, additive effects. The convergent trend resulting from combinations of factors increasing host-resistance is shown in Fig. 1.

The differences between the reciprocal hybrid groups reflect the action of a maternal influence, here presumed to be identical with the milk-factor. In all

FIG. 1.—Diagram showing the median lethal doses (scale on left) and their standard errors (vertical lines) for the C3H carcinoma irradiated in various situations. Abscissae: Degrees of genetic difference between host and tumour arbitrarily spaced so as to emphasise the convergent trend towards lower effective dosage as increased host-resistance is evoked.

experiments testing immunogenetic differences, the factor-free hybrid diverges most from the C3H parent, while its factor-harbouring counterpart consistently shows an intermediate and somewhat more variable effect. The milk-factor, acting on the infant hybrid mouse, thus seems partially and inconstantly to obliterate immunogenetic differences, virtually inducing an acquired tolerance to the tumour, analogous to the "actively acquired tolerance to foreign cells" described by Billingham, Brent and Medawar (1953) in CBA mice.

The results of the foregoing series of experiments lend strong and consistent support to the hypothesis that the radiosensitivity of a tumour is a function of a specific systemic host resistance against that tumour. This single assumption suffices to explain four observations bearing upon the problem:

(1) That the tumour growing in hybrids is more radiosensitive than in parent-strain hosts;
(2) the milk-factor, tending to obliterate immunogenetic reactions to the tumour, reduces its radiosensitivity;
(3) tumours arising from radiation-attenuated implants in the C3H host are more radiosensitive (" induced radiosensitization");
(4) whole body irradiation, by deranging the resistance mechanism of the host, reduces the radiosensitivity of the tumour (Cohen and Cohen, 1953*b*).

If, as seems indicated by the present investigation, an irradiated tumour fragment is subjected at the time of implantation to a reaction on the part of the host approaching a certain well-defined maximum limit of intensity, and mediated through the same underlying mechanism as immunogenetic resistance, then the following three additional observations are also seen to fit the original hypothesis:

(5) The lethal dose for irradiation prior to implantation is smaller than that required by the established tumour *in situ*;
(6) the lethal dose for irradiation prior to implantation is not affected by genetic or maternal factors;
(7) radiosensitization induced by prior attenuation is less marked in hybrid hosts.

### SUMMARY.

When the C3H mammary carcinoma was irradiated before implantation into reciprocal $F_1$ hybrid hosts, the $LD_{50}$ was found to be 2700 r in (CBA × C3H)$F_1$ mice, presumably factor-free, and 3000 r in the factor-harbouring (C3H × CBA)$F_1$ reciprocal. These values do not differ significantly from each other or from that of the same tumour in the C3H parent-strain host (2800 r).

Whereas tumours arising from radiation-attenuated, viable implants in the parent strain had shown an increased radiosensitivity when subsequently treated *in situ*, the median lethal dose for similarly attenuated tumours in $F_1$ hybrid hosts did not differ significantly from non-attenuated homoplasts. The factor-harbouring hybrid, however, bore a closer resemblance to the C3H parent, showing a somewhat greater tendency to induced radiosensitization than the factor-free reciprocal.

It is concluded that radiosensitivity is a function of host resistance which may be enhanced both by inoculation of radiation-attenuated implants, and by immunogenetic differences between the heterozygous hybrid host and the homozygous tumour, but a combination of both procedures, which presumably act through the same underlying mechanism, has no obvious additive effect.

We are indebted to Dr. J. F. Murray for providing facilities at the South African Institute for Medical Research where the C3H/Cg mouse colony is maintained. We are also grateful to Mrs. Shelley Jacobson for invaluable skilled assistance in the upkeep and breeding of this colony.

### REFERENCES.

BILLINGHAM, R. E., BRENT, L., AND MEDAWAR, P. B.—(1953) *Nature*, **172**, 603.
COHEN, A., AND COHEN, L.—(1953*a*) *Brit. J. Cancer*, **7**, 231—(1953*b*) *Ibid.*, **7**, 452.—(1954*a*) *Ibid.*, **8**, 303.—(1954*b*) *Ibid.*, **8**, 313.
FINK, M. A., SNELL, G. D., AND KELTON, D.—(1953) *Cancer Res.*, **13**, 666.
GORER. G.—(1947) *Ibid.*, **7**, 634.